# Cistrome Data Browser: integrated search, analysis and visualization of chromatin data

**DOI:** 10.1093/nar/gkad1069

**Published:** 2023-11-16

**Authors:** Len Taing, Ariaki Dandawate, Sehi L’Yi, Nils Gehlenborg, Myles Brown, Clifford A Meyer

**Affiliations:** Center for Functional Cancer Epigenetics, Dana-Farber Cancer Institute, Boston, MA, USA; Department of Data Science, Dana-Farber Cancer Institute, Boston, MA, USA; Department of Biomedical Informatics, Harvard Medical School, Boston, MA, USA; Department of Biomedical Informatics, Harvard Medical School, Boston, MA, USA; Center for Functional Cancer Epigenetics, Dana-Farber Cancer Institute, Boston, MA, USA; Department of Medical Oncology, Dana-Farber Cancer Institute, Brigham and Women's Hospital, and Harvard Medical School, Boston, MA, USA; Department of Data Science, Dana-Farber Cancer Institute, Boston, MA, USA; Department of Biostatistics, Harvard T.H. Chan School of Public Health, Boston, MA, USA

## Abstract

The Cistrome Data Browser is a resource of ChIP-seq, ATAC-seq and DNase-seq data from humans and mice. It provides maps of the genome-wide locations of transcription factors, cofactors, chromatin remodelers, histone post-translational modifications and regions of chromatin accessible to endonuclease activity. Cistrome DB v3.0 contains approximately 45 000 human and 44 000 mouse samples with about 32 000 newly collected datasets compared to the previous release. The Cistrome DB v3.0 user interface is implemented as a single page application that unifies menu driven and data driven search functions and provides an embedded genome browser, which allows users to find and visualize data more effectively. Users can find informative chromatin profiles through keyword, menu, and data-driven search tools. Browser search functions can predict the regulators of query genes as well as the cell type and factor dependent functionality of potential *cis-*regulatory elements. Cistrome DB v3.0 expands the display of quality control statistics, incorporates sequence logos into motif enrichment displays and includes more expansive sample metadata. Cistrome DB v3.0 is available at http://db3.cistrome.org/browser.

## Graphical Abstract

**Graphical Abstract:**
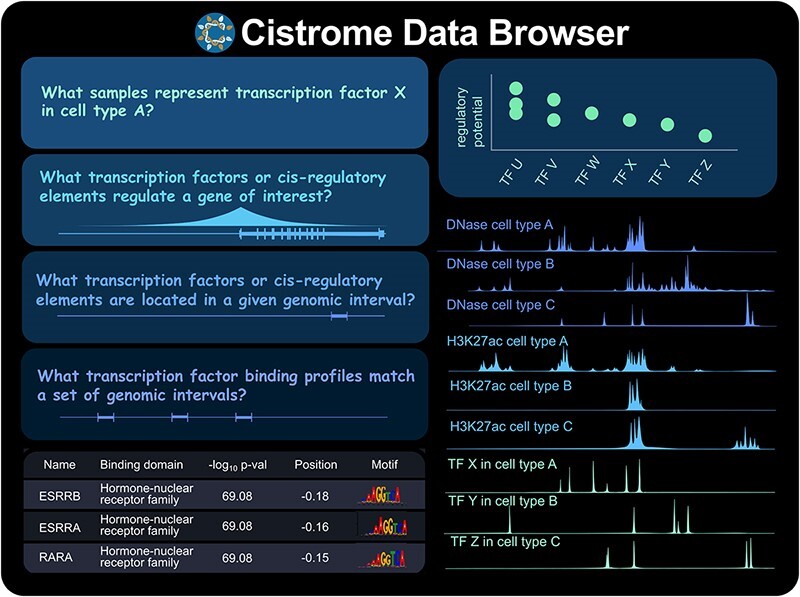

## Introduction

The regulation of genes in humans and mice involves a highly complex interplay between RNA polymerase, transcription factors and cofactors, chromatin remodeling complexes, histone readers, writers and erasers, the histone modifications themselves, as well as chromatin structural elements (1,2). Cistromes, defined as genome-wide maps of the *cis-*regulatory binding sites of trans-acting factors, serve as invaluable tools for elucidating the intricate biology of gene regulation (3–6). The Cistrome Data Browser was developed to provide the biomedical research community with easier access to chromatin-related data and analysis (5,7). This valuable resource incorporates a wide array of data types, including ChIP-seq (8–11), DNase-seq (12,13) and ATAC-seq (14), which have found widespread adoption in biomedical research, particularly in the study of gene regulation mechanisms. In addition to large-scale initiatives like ENCODE (15) and the NIH Epigenomics Roadmap (16), numerous individual laboratories have contributed high-quality chromatin data to the NCBI Gene Expression Omnibus (GEO) (17). It can be challenging to compare or integrate data from consortia and various research groups, as the metadata annotations, processing, and quality control procedures are not standardized. To mitigate this problem, the data in the Cistrome Data Browser are processed using standardized pipelines that annotate, determine quality control metrics, extract signal, and carry out several core analyses that facilitate the understanding of gene regulation. Other databases and websites collecting and integrating chromatin related data include ChIP-Atlas (18), ChIPBase (19) and ReMap (20). Cistrome DB differs from these in terms of sample coverage, comprehensive quality control metrics, data browsing and querying capabilities, data visualization, and downstream analysis functions and methodology.

Cistrome Data Browser v3.0 introduces an improved user interface that streamlines both menu-driven and text-based search modalities, and offers data-driven search capabilities. Furthermore, the new version allows users to visualize genome track data using an embedded Gosling browser display (21). The new version incorporates additional processed data, more comprehensive metadata annotations, and detailed quality control reports, making it a more powerful tool for researchers studying chromatin, gene-regulation and the non-coding part of the genome.

## Materials and methods

### Data collection

ChIP-seq, DNase-seq and ATAC-seq samples were identified in the public databases: NCBI Gene Expression Omnibus (GEO) (17), Encyclopedia of DNA Elements (ENCODE) (15), and Roadmap Epigenomics Project (16). In the case of GEO, all sample identifiers were obtained from the SRA database (22). Sample XML files were downloaded from GEO.

### Metadata annotation

The sample XML files were parsed to determine the species and data type. Cell and tissue type annotation was done using a version of the MetaSRA RNA-seq annotation package (23) that we modified and adapted for chromatin sample annotation. Cell or tissue type annotation were derived from multiple biomedical ontologies including Cellosaurus (24), Cell Line Ontology (25), Cell Ontology (26), Experimental Factor Ontology (27) and Uberon (25). Transcription factors and chromatin regulators were annotated according to official gene symbols. Antibody catalogue identifiers were used in the parser as an additional means of identifying target factors in ChIP-seq data. Cistrome DB v3.0 often provides multiple annotations for a given sample. Each annotation is given a score to indicate the confidence of the annotation.

### Data processing and quality control

**Figure 1.**
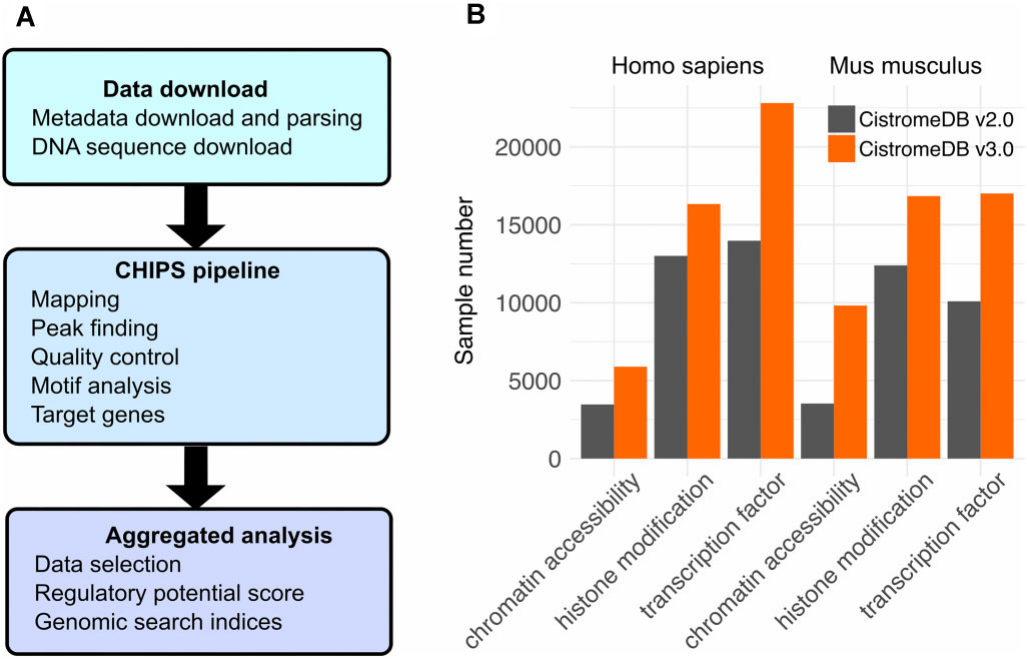
Data processing and statistics. (**A**) Cistrome DB v3.0 processing pipeline and (**B**) processed data statistics for Cistrome DB v2.0 and v3.0.

To ensure consistency of Cistrome DB data, raw DNA sequence data for each sample was downloaded and uniformly processed by the CHIPS pipeline (28), implemented in the Snakemake (29) workflow language (Figure 1A). SRA files were obtained from NCBI and FASTQ files were extracted using the fastq-dump software. The CHIPS pipeline implements the same processing as applied the earlier version of Cistrome DB (5,6): BWA (30) for mapping reads to the hg38 or mm10 genomes; MACS2 (31) for identifying statistically significant peaks; SeqPos for motif enrichment in transcription factor or chromatin regulator ChIP-seq samples (32); and regulatory potential models for target gene identification (33).

Cistrome DB v3.0 quality controls include read count, mappable read count, sequencing quality, PCR amplification artifacts, numbers of peaks found, proportions of peaks in chromatin accessible regions, proportions of reads in peaks and evolutionary conservation scores. Details of these quality controls can be viewed under the Sample Details pages and information about the process is available on the Cistrome DB v3.0 website. To allow users to assess sample quality we display three informative and complementary statistics on the sample table pages as green or red dots to indicate higher or lower quality, respectively. For consistency, cutoffs are the same as those used in Cistrome DB v2.0 (7). Although Cistrome DB v3.0 also includes some low-quality samples from the GEO repository, these samples may nevertheless contain useful information that is complementary to other samples in the database.

### Gene regulator search

Cistrome DB v3.0 incorporates three ‘toolkit’ functionalities that were previously developed (7). These functions can now be accessed directly on the main Cistrome DB browser page. For the gene regulator search, assignment of TFs to genes is based on regulatory potential (RP) scores that reflect the collective influence of the binding sites of a given TF on genes nearby these sites and assume that TF binding sites near the TSS are more likely to regulate the gene than those further away. The RP score for gene $i$ and transcription factor $j$ is defined as: ${R}_{ij\ } = \ \mathop \sum \limits_{{\mathrm{peak}}\ k{\mathrm{\ of\ TF}}\ j} {2}^{ - {x}_{ijk}/\Delta }$, where $\Delta$ is the decay distance and ${x}_{ijk}$ is the genomic distance between peak $k$ of TF $j$ and the TSS of gene $i$. As different TFs might regulate genes over different ranges of genomic influence, and different genes can be influenced by enhancers over different ranges (34), RP scores are calculated for each TF and gene using short (1 kb), mid-range (10 kb) and long-range (100 kb) decay distances. To focus on high quality and high confidence peaks, only peaks with 5-fold enrichment over background were used in these RP score calculations. As the total number of peaks varies between samples and this number influences the RP scores, the RP scores for each sample were standardized to fit into a range between 0 and 1 to enable cross-sample comparison. In the web interface, users can input a coding gene name and select the required parameters (species, distance). The Cistrome DB v3.0 target gene search queries RP scores across samples and returns samples, ranked based on the RP score for the query gene.

### Genomic interval search

In the ‘genomic interval’ and ‘genomic interval set’ search modes, GIGGLE (35), an efficient search engine for large-scale genomic loci is used to compare the user-defined interval or set of intervals with Cistrome DB peaks. Only peaks with fold-enrichments to background that are >5 are included in the GIGGLE index. We built GIGGLE indices using 1000 and 10 000 peaks with the highest fold-enrichments to provide options that keep running time low while without compromising results.

### Website implementation

The website implementation includes a backend in which metadata and quality control data are stored in a MySQL database which can be queried via REST API implemented with Django 3.2 and the Django REST framework. The Cistrome ‘toolkit’ data for data- driven searches is saved in HDF5 and GIGGLE formats and accessed by REST API. Signal tracks are stored in BigWig format, extracted using USCS BigWig reader tools (36). The client software is implemented in React, incorporating the Gosling browser (21), Visx (airbnb.io/visx) and D3 (d3js.org) for data-driven search displays, and LogoJS (weng-lab.github.io) for motif logo displays.

## Results

**Figure 2.**
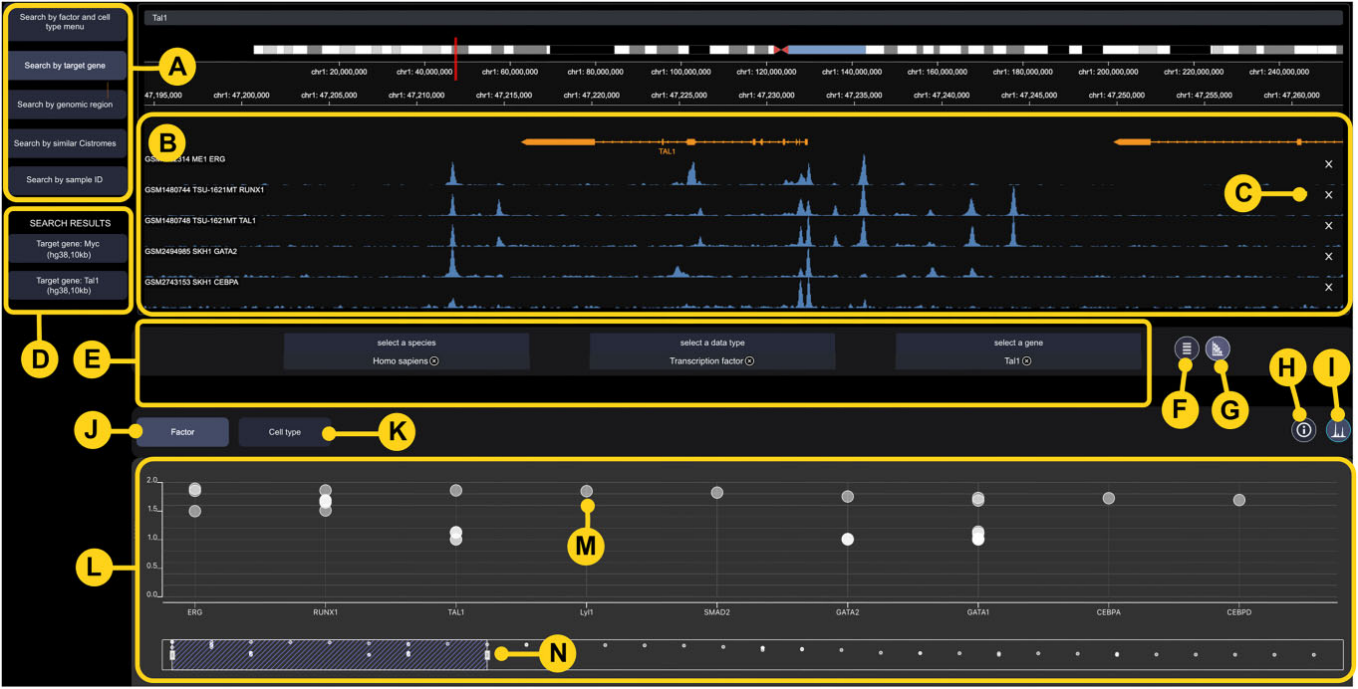
Cistrome DB v3.0 user interface. (**A**) search mode menu, (**B**) Gosling browser (**C**) signal track close button, (**D**) search results history, (**E**) search query parameter selection, (**F**) button to show results in a tabular format, (**G**) button to show results in a chart format, (**H**) button to show selected details of sample metadata, quality controls, target genes and motifs, as well as further options, (**I**) button to show selected sample signal track in Gosling browser, (**J**) button to select view of results by factor in chart, (**K**) button to view results by cell type, (**L**) chart view of results from data driven searches, (**M**) button to select a sample and either display detailed information or signal track, (**N**) bar for selecting set of sample to display in chart.

Cistrome DB v3.0 contains approximately 30 000 new samples, with metadata, quality control and down-stream analysis (Figure 1B). Chromatin accessibility samples have seen a substantial increase, thanks to the widespread adoption of ATAC-seq. Cistrome DB v3.0′s interface is now a single-page application, seamlessly integrating multiple search and visualization features (Figure 2). This interface allows users to find data through keyword, menu and data-driven search modalities, select from the discovered data, and display tracks on the embedded Gosling genome browser.

The data-driven search modalities, target gene, genomic interval, and set of genomic regions, previously implemented as the ‘Cistrome Toolkit’, are now incorporated in a unified analysis interface (Figure 2A). The target gene search addresses the question: ‘What factors regulates a gene of interest?’ This function provides a list of transcription factors in the database that are most likely to regulate a query gene, based on a regulatory potential model. The search can also be used to find chromatin accessibility and histone modification data relevant to the gene. The genomic region search modality answers the question: ‘What factors bind in a genomic interval of interest?’ This function identifies TF binding, histone modifications, and chromatin accessibility in any query genomic interval shorter than 1Mb. The genomic region set modality allows users to upload a set of genomic intervals in BED file format and find samples that have similar genome regions.

The search by menu allows users to dynamically filter by transcription factor or histone mark on the one hand, and tissue and cell type ontology on the other. Samples with metadata annotation matching the query are displayed in a table with three informative quality control metrics, 10-fold enriched peak count, fraction of reads in peaks (FRiP), fraction of peaks in a DNase-hypersensitive regions (derived from a variety of cell types). Results from the data-driven searches can be displayed in either table of chart format (Figure 2L). Detailed sample information and signal tracks can be viewed through mouse interactions with either the table (Figure 2F) or the chart (Figure 2G). Samples tracks can be loaded (Figure 2I) into the embedded Gosling genome browser (Figure 2B), that allows for smooth zooming and panning. Results from previous searches in a session, are saved in a menu (Figure 2D) which allows users to quickly access results from multiple searches and compare tracks on the genome browser.

**Figure 3.**
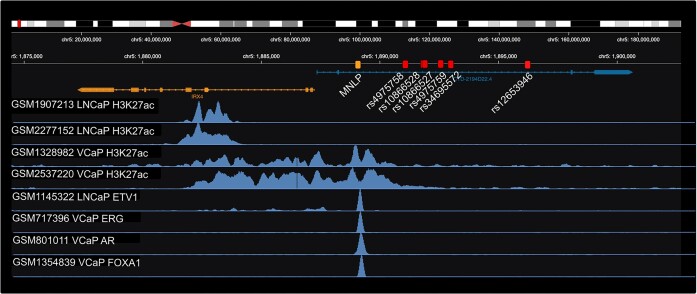
Prostate cancer risk locus analysis using Cistrome DB chromatin profiles. Prostate cancer SNP risk loci are shown as red boxes, and a multi-nucleotide polymorphism (MNLP) is shown in orange. H3K27ac shows active enhancer and promoter activity in LNCaP and VCaP prostate cancer cell lines, which are homozygous for short and long MNLP alleles, respectively. Several transcription factor binding sites coincide with the MNLP.

As a case study we show how Cistrome DB v3.0 can be used to facilitate the investigation of gene regulatory mechanisms in cancer. The 5p15.33 prostate cancer risk locus (Figure 3) is a small (∼6 kb) region harboring six prostate cancer associated SNPs in linkage equilibrium with each other, 7 kb upstream of the Iroquois Homeobox 4 (IRX4) promoter (37–39). Histone 3 lysine 27 acetylation (H3K27ac) is a marker of activate enhancers. Using the Cistrome DB *ontology and factor search* we identify high quality H3K27ac tracks from LNCaP and VCaP prostate cancer cell lines and upload signal tracks into the *genome browser for visualization*. When using the track browser, we observe that none of the GWAS SNPs show enhancer activity in LNCaP or VCaP cell lines. *Search by genomic interval* applied to the genomic interval that includes the SNPs and the IRX4 promoter, identifies TF ChIP-seq peaks in prostate cancer cell lines, which are then be displayed on the data browser. This reveals binding sites for several known prostate cancer regulatory TFs, including AR, ERG, ETS1 and FOXA1 in a putative enhancer region that does not belong to the set of GWAS SNPs. In addition to the GWAS SNPs, a multiple nucleotide length polymorphism (MNLP) near the IRX4 promoter has also been implicated in prostate cancer susceptibility (39,40). The MNLP has two alleles: a short one (21 bp) and a longer one (47 bp). From the Cistrome DB tracks we can see that the candidate enhancer region coincides exactly with the MNLP genomic position. Spisak *et al.* (39) showed that a single copy knock-in of the long allele in LNCaP cells, which are homozygous for the short allele, facilitates binding of the androgen receptor (AR) along with increased H3K27ac, and 3-fold upregulation of IRX4 gene expression. VCaP is homozygous for the long allele, which is consistent with the H3K27ac activity differences between LNCaP and VCaP. Using the Cistrome DB v3.0 integrated search features, in combination with the browser, allows users to develop and refine hypotheses and to identify data sets that can be used for more in depth analyses.

## Discussion

Cistrome DB v3.0 provides new data and an integrative interface that will help researchers make better use of publicly available human and mouse chromatin data.

Combining text-driven and data-driven searches with chromatin track visualizations will empower investigators to augment their knowledge of chromatin biology with the large-scale collection of chromatin data. Future updates of the Cistrome DB will incorporate single cell data, offer additional search options and enhance visualizations of chromatin landscapes.

## Data Availability

Cistrome DB v3.0 is freely available at http://db3.cistrome.org/browser.
